# A professional training simulator for skill acquisition in ultrasound-guided lumbar facet syndrome intervention: design and educational evaluation

**DOI:** 10.3389/fdgth.2026.1761690

**Published:** 2026-03-06

**Authors:** Belén Curto, Vidal Moreno, Juan-Alberto García-Esteban, David Sanchez-Poveda, Pablo Alonso, Felipe Zaballos

**Affiliations:** 1 Department of Computer Science and Automation, University of Salamanca, Salamanca, Spain; 2 Department of Anesthesiology, Assistential University Complex of Salamanca (CAUSA), Salamanca, Spain

**Keywords:** computer-based US simulators, facet syndrome treatment, interventional ultrasonography, life-like HMI devices, medical training

## Abstract

**Introduction:**

Ultrasonography (US) plays a central role in modern diagnostic and interventional medicine, particularly in the management of facet-origin chronic low back pain, a highly prevalent condition in industrialized societies. However, its clinical effectiveness depends largely on the level of specialist training, requiring advanced skills in probe manipulation, sonoanatomy interpretation, brain-hand-eye coordination, and safe planning of interventional procedures. This work presents the development of a training simulator for ultrasound-guided treatment of lumbar facet syndrome; the simulator is implemented within a modular learning framework designed to support the flexible and efficient creation of procedure-specific simulators.

**Methods:**

The developed simulator integrates a physical replica of an ultrasound probe, enabling trainees to practice realistic handling. Probe movements performed by the trainee along the scan path are continuously tracked and mapped to corresponding ultrasound images and videos, previously acquired by clinical experts from a real subject and displayed in real time on a computer screen. For interventional planning, a virtual syringe-and-needle component allows trainees to simulate needle orientation and insertion depth, with relevant anatomical structures highlighted as visual learning aids.

**Results:**

A validation study was conducted involving 18 final-year medical students using an ad hoc questionnaire addressing usability, realism, learning support, and overall training experience. The results demonstrate a high level of student acceptance and a positive perceived impact on the acquisition of skills related to ultrasound-guided exploration and interventional planning. Most students reported accelerated skill acquisition in US examination (89% very satisfied, 11% satisfied) and high motivation (83% very satisfied, 17% satisfied). Overall performance and the likelihood of recommending the simulator received the highest rating from all participants (100%).

**Discussion:**

From the perspective of students, the simulator provides a realistic and supportive learning experience, particularly due to the realism of the physical probe replica, the quality of the graphical user interface, and the guided learning process. From the perspective of instructors, the effectiveness of the simulator depends on the quality of the learning resources and the scope of the training cases. Although the preparation and curation of high-quality ultrasound datasets and annotations remains time-consuming, the framework significantly facilitates and adds flexibility to the development of new case studies. This positions the approach as a valuable complementary training resource, helping to bridge the gap between theoretical instruction and supervised clinical practice in ultrasound-guided procedures.

## 1 Introduction

The medical community is continually confronted with the challenge of replacing conventional clinical examination, diagnostic, and therapeutic procedures with alternative approaches that enhance patient safety and comfort while reducing associated healthcare costs [1]. In this context, medical imaging has progressively assumed a central role in contemporary medicine, substantially transforming both diagnostic and therapeutic clinical practices [2]. Procedures such as tissue sampling for biopsy, injection of anesthetic agents, or implantation of radioactive sources for brachytherapy require the accurate percutaneous insertion of a needle into a precisely defined region of soft tissue; this process can be effectively guided by imaging modalities [3]. Addressing this challenge requires healthcare professionals to adapt to innovative techniques and integrate state-of-the-art technologies into routine patient care.

In recent years, ultrasonography (US) has been increasingly adopted for both diagnostic and interventional imaging procedures [2]. This expansion can be attributed to its favorable radiological safety profile [4], high patient tolerability, and reduced procedural times [3]. In contrast, other imaging modalities, such as conventional radiography or computed tomography (CT), are generally reserved for follow-up examinations or more complex diagnostic evaluations. Furthermore, US equipment is portable and cost-effective [1]. However, image quality is constrained by factors including a low signal-to-noise ratio, modality-specific artifacts, reduced spatial resolution in deeper anatomical regions, and the inability of ultrasound waves to propagate through air or osseous structures [5], among other technical limitations. Despite these limitations, the use of ultrasound has become widespread across multiple medical specialties and is increasingly being incorporated as a routine diagnostic tool in primary care settings [6]. According to Chen et al.[7], the portable ultrasound scanner is considered the “visual stethoscope” of the 21st century.

However, its clinical effectiveness ultimately depends on the level of training and expertise of the operator [1, 8]. In this context, specialists must first acquire the technical skills required to manipulate the ultrasound probe, including 3D displacements and orientations, to accurately acquire and visualize anatomical structures while minimizing image artifacts [9]. This process requires appropriate brain–hand–eye coordination [10, 11]. Second, visual training is essential for accurate identification and interpretation of anatomical structures in ultrasound images (sonoanatomy) [9]. Third, in interventional procedures specific to each specialty [ultrasonosurgery [3]], which require needle insertion into target soft tissues, the development of adequate motor coordination and precise instrument control is vital [3, 10].

One of these procedures is infiltration, which has been used for decades in pain management using established anatomical landmark-guided techniques. More recently, ultrasound-guided infiltration has gained considerable clinical acceptance, as it enables accurate drug deposition at the target structure [12] and real-time monitoring of the injected volume. This procedure is currently applied both for the diagnosis and treatment of low back pain. In industrialized societies, it is estimated that between 70% and 80% of the population will experience low back pain at least once during their lifetime, and among these cases, a substantial proportion will progress to chronic low back pain [13], representing a major public health concern. Among the anatomical structures involved in chronic low back pain are the lumbar facet joints, whose degeneration or functional alteration may lead to the so-called lumbar facet syndrome.

In clinical practice, the diagnosis of facet-origin pain is primarily based on diagnostic blocks, which are among the most widely used methods for identifying the pain source. During diagnostic blocks, local anesthetics are injected into the joint to assess pain relief and confirm the involvement of the targeted structure. Once the diagnosis is confirmed, minimally invasive interventional procedures with therapeutic purposes may be performed, such as intra-articular steroid injections or medial branch blocks. Compared with conventional surgical techniques, these procedures are associated with lower morbidity. However, they require a high degree of precision in instrument handling due to the lack of direct visualization of the working area and the proximity of vital anatomical structures. Consequently, comprehensive theoretical and practical training is required to achieve the level of motor coordination and spatial accuracy necessary for these interventional procedures, as an improper needle trajectory may result in unintended damage to anatomical structures along the path to the target tissue [10, 12].

In invasive therapeutic procedures, practice involving human volunteers is considered unacceptable. Consequently, alternative training strategies are required [14]. Recently, medical simulation has positioned itself as a fundamental tool for professional training [15, 16]. Within the health sciences, simulation techniques are defined as the use of devices, systems, or controlled environments, called simulators, designed to reproduce real clinical conditions for the purpose of performing specific practices [17]. Through these practices, technical and cognitive skills are acquired, facilitating a safe and accelerated learning process [15].

Tissue-simulating phantoms are among the most widely used training simulators in the field of medical imaging [11]. These phantoms are physical mannequins designed to reproduce specific regions of human anatomy and to mimic the acoustic properties of real biological tissues [11, 18]. They are imaged using clinical ultrasound transducers [19, 20]. However, such phantoms often exhibit limited anatomical realism and, more notably, reduced durability when subjected to repeated needle punctures [21].

Computer-based US simulators can replicate realistic clinical experiences by generating carefully designed, structured scenarios in which user interaction is guided and controlled [22]. These computer-based US simulators can be classified [17] according to the user interaction devices employed, namely, the human–machine interface (HMI). Trainees can interact using basic input devices, such as a mouse and keyboard, to manipulate virtual graphical elements displayed on the screen [23, 24]. Alternatively, more advanced simulators incorporate dedicated interaction devices intended to replicate those used in real clinical practice, including ultrasound probes and needles, both for diagnostic exploration and interventional procedures [25]. In this latter group, a higher degree of user immersion in the learning environment is achieved. However, accurate 3D localization and tracking of the interaction devices [17], such as the US probe, are required.

This paper presents the design and educational validation of a simulator for ultrasound-guided intervention in lumbar facet syndrome, specifically developed for professional training purposes. The simulator was constructed using a conceptual framework that provides a structured approach for creating procedure-specific simulators across multiple clinical specialties that utilize ultrasound imaging, thereby addressing the common problem of duplication of development efforts [26]. The developed simulator incorporates a realistic sensorized replica of an ultrasound probe that enhances brain–hand–eye coordination of the trainees, as the perception of probe movement and the corresponding visual feedback closely recreate sensations experienced in real clinical practice. To assess whether this simulator offers an authentic user experience that promotes cognitive learning of ultrasound-guided interventions for lumbar facet syndrome, a comprehensive validation study was conducted with final-year medical students, additionally evaluating fundamental aspects of human interaction with the proposed technological environment.

## 2 Materials and methods

This section begins with a description of the conceptual design underlying the proposed training framework, followed by a detailed presentation of its main components. Subsequently, the lumbar facet syndrome treatment simulator, developed using this framework is described. Finally, the methodology used to evaluate the simulator is presented.

### 2.1 Conceptual overview of the learning framework

Most malpractice related to ultrasonography is attributed to misinterpretation of images resulting from insufficient skill and training [27]. Therefore, future specialists must have at their disposal powerful technical resources that allow them to practice, first, US-guided anatomical exploration and, second, US-guided interventional procedures.

A team of five medical experts, who also serve as educators, identified the essential skills required for future specialists in US-based interventions. These skills include US image acquisition, anatomical interpretation of US images (sonoanatomy), needle path planning and brain–hand–eye coordination.

Based on these prerequisites, the proposed learning framework integrates the following theoretical and practical resources: a US image-based simulator for musculoskeletal exploration and diagnosis (US-DES), a US treatment simulator (US-TS), a 3D anatomical model, and a complete set of multimedia tutorials. This framework enables the flexible and efficient design and development of simulators for practicing specific US-guided interventional treatments, such as facet joint infiltration.

**Figure 1:**
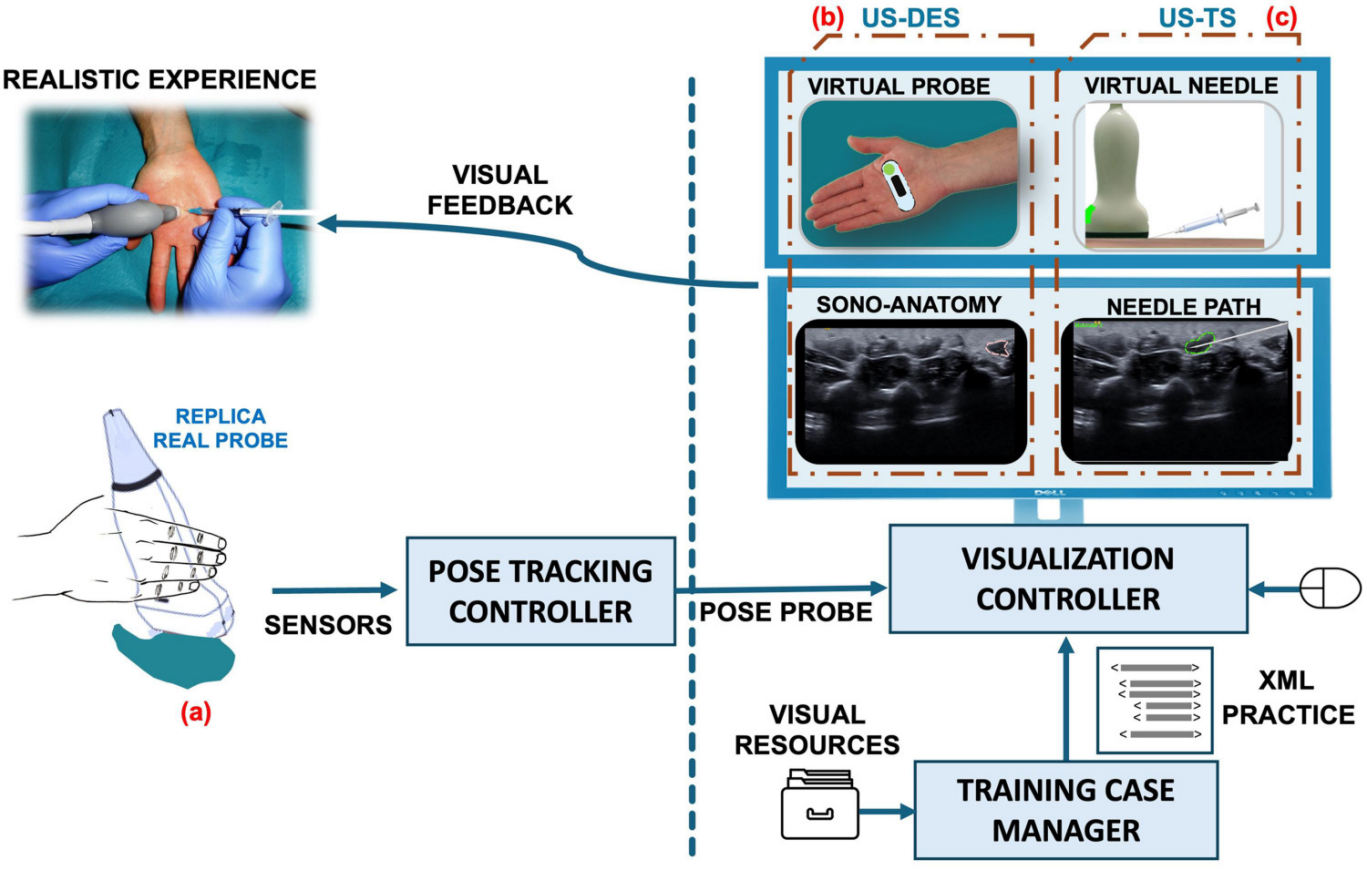
Devices and modules at the simulator for the recreation of realistic experiences. (a) US Probe Real Replica, (b) UD-DES, (c) US-TS.

Conceptual aspects related to specific treatments and sonoanatomy are addressed through the tutorial lessons, supported by multimedia resources and a powerful visual didactic component, including a complete 3D anatomical model. Practical training follows a teaching-learning strategy based on case analysis, designed by a team of health experts. The student can practice these case studies using the two simulators (Figure 1), which constitute the core components within this framework.

Using the US-DES simulator, the student can train in US image acquisition, recognize the spatial correspondence between the anatomical features observed in the US image and the overall 3D representation of the patient anatomy, and develop hand–eye coordination for the visualization of musculoskeletal structures. The distinctive feature of the US-DES simulator, compared with existing computer-based US simulators, is the incorporation of a realistic sensorized replica of a US probe (Figure 1a) that the trainee can manipulate in the same manner as a real probe. Specifically, the student can move the probe on a surface along a *planar scan path* and then perform rotational movements around the three axes along a *rotation scan path*. These movements are captured by the sensors embedded in the replica probe. Then, the *Virtual Probe* window on the computer (Figure 1b) reproduces these movements in real time by displaying a virtual probe over a photograph of the human model corresponding to a given case study. Simultaneously, the *Sonoanatomy* window displays the US images according to the pose (position and orientation) of the replica along a *planar scan path* or a *rotation scan path*.

Using the US-TS simulator, the student can train in US-guided interventional procedures to develop practical skills related to planning the needle path toward the anatomical target, referred to as the *needle insertion path*. Two virtual components will be displayed in the *Virtual Needle* window of the computer interface: a syringe and a needle (Figure 1c). The trainee manipulates the syringe using the computer mouse, where they can modify the syringe orientation and, consequently, the needle orientation. In the same manner, the trainee can change the depth of needle penetration. In the *Needle Path* window, the needle is displayed on a US video of the treatment area, reflecting the selected orientation and penetration depth. As a learning aid, the musculoskeletal structures of interest are highlighted on the US images. In this way, the student can visually check whether access to the target structure is feasible along a given *needle insertion path*, with the orientation of the syringe-needle assembly fixed.

### 2.2 Learning framework architecture and components

This section first describes the training methodology and data management, then the physical ultrasound probe replica, and finally the three main software components that integrate the learning framework.

The training methodology is based on case studies, with data management implemented through eXtensible markup language (XML) files. A physical replica of the US probe, developed by our research team [28], and equipped with MEMS sensors to capture its 3D pose, is briefly described.

Regarding the software components, the *Pose Tracking Controller* is responsible for estimating the 3D pose of the replica in real time to track the movements performed by the student. The *Visualization Controller* reproduces these movements on the computer screen via a virtual probe and accesses a repository of prerecorded US images, which are displayed according to the probe movements. Since a complete set of case studies must be developed, the learning framework includes a high-level app, *Training Case Manager*, which enables teachers to recreate any medical scenario that uses US technology.

#### 2.2.1 Training methodology and data management

The teaching-learning methodology is based on case analysis. Therefore, medical experts in the specialty targeted by each simulator are responsible for designing and building a set of practical cases. First, teachers should prepare a repository of visual resources, including photographs of a human model, ultrasound images, and illustrations of anatomical slices corresponding to the study cases.

Within the learning framework, a file-based data model using XML is employed to enable the flexible definition of the case studies. XML is a markup language that defines a set of rules for document coding. Its main strength is that it allows structured data to be managed and shared among different systems, applications, and organizations through a human-readable text format, while also providing semantic meaning to the stored information. A fundamental advantage of this XML-based data model is that training practices can be added, modified, or deleted without modifying the software source code.

**Figure 2:**
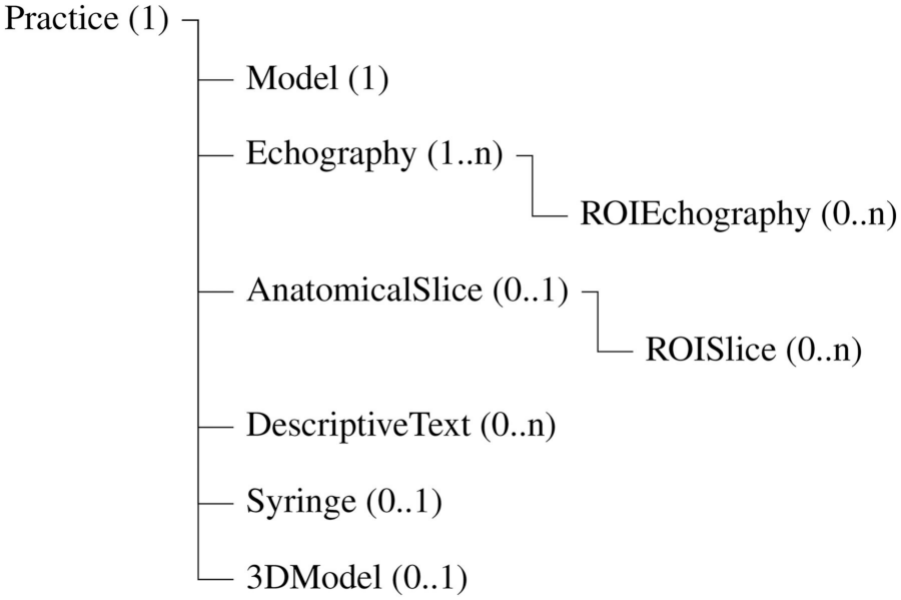
Conceptual hierarchical structure of the XML files.

In the XML files, our own set of rules is defined to configure all the resources required for each anatomical exploration practice and the treatment procedure in the real clinical scenario. Each XML file consists of a list of elements (Figure 2) and attributes organized in a hierarchical structure according to the semantics of the case study. Specifically, a root element named *Practice* contains the following eight elements:

*Model:* It defines the human model and the scan path, including attributes such as the filename of the model image, display size, initial virtual probe orientation and the set of points defining the scan path.
*Echography:* It specifies the stored real US images and their format. These US images are displayed during the scan path execution, including those corresponding to the correct treatment sites, as well as the US video on which the needle is overlaid during the treatment. Its attributes include the US image identifiers, the target treatment area, the surrounding area provided as a training aid, the needle entry point, the depth of the US images, and the video sequences displayed at the correct treatment site in both conventional and Doppler modes.
*ROIEchography:* It defines the regions of interest (ROIs) on the US image associated corresponding to the relevant anatomical structures of the case study. Multiple *ROIEchography* elements may be associated with a single *Echography* instance. Its attributes specify the polygonal ROI contour and the anatomical structure identification parameters (e.g., type, name, color, and font).
*AnatomicalSlice:* It specifies the resources associated with the anatomical slice illustration, enabling students to relate real anatomical structures to those visualized on the US image, including the illustration filename and the display format.
*ROISlice:* It specifies the ROIs on the anatomical slice illustration, analogous to *ROIEchography* for the US image.
*DescriptiveText:* Associated with each ROI defined on the US image and the *AnatomicalSlice*, this element provides a detailed explanation of each anatomical structure of interest. In addition to standard visualization attributes, it includes anatomical description attributes (e.g., function, innervation).
*Syringe:* It defines the syringe attributes for the simulated procedure, including its virtual image, the reference virtual probe image, the initial puncture point, and the insertion side relative to the ultrasound probe.
*3DModel:* It configures the neuromuscular systems and the anatomical structures to be displayed in the 3D anatomical model for a given practice.

#### 2.2.2 Physical US probe replica

Realistic user immersion in the training technological environment is achieved through a physical replica of a real transducer, developed by our research team [28]. Motion sensing in the replica is performed using MEMS-based sensors, such as an inertial measurement unit (IMU) and an optical mouse chip. The IMU data enable estimation of the attitude (3D orientation) of the probe as the trainee performs rotational movements along a *rotation scan path*. The optical mouse chip enables the detection of 2D linear displacements as the student performs movements along a *planar scan path*.

All sensors are housed within a plastic shell, built via additive manufacturing and designed to closely replicate the geometry and ergonomics of a real US probe.

#### 2.2.3 Pose Tracking Controller

Along a *rotation scan path*, the *Pose Tracking Controller* is responsible for estimating the three angular orientation coordinates of the physical replica in real time to track the movements performed by the student. To this end, it acquires data from the different IMU sensors and fuses these measurements using an extended Kalman filter-based algorithmic approach to reduce estimation uncertainty. It periodically sends the computed 3D orientation to the *Visualization Controller*.

Along a *planar scan path*, the forward–backward and left–right linear movements of the probe are converted by the optical mouse sensor into displacement signals. These signals are processed by the operating system services of the desktop computer, allowing the real 2D linear displacements performed with the probe replica to be faithfully reproduced on the computer screen.

#### 2.2.4 Training Case Manager

To facilitate XML file configuration by teaching experts, the technical support team developed a graphical user interface (GUI)-based application called *Case Training Manager*. This user-friendly tool enables instructors to create and manage scase studies without requiring knowledge of XML encoding. Through the GUI, instructors can browse the visual resource repository, specify visualization properties and scan paths, and annotate anatomical structures of interest directly on images.

The generated XML files and associated multimedia resources are organized into case-specific repositories and are later processed and interpreted by the *Visualization Controller* during training sessions. *Case Training Manager* was implemented in C++ using the Qt cross-platform framework, ensuring compatibility with major operating systems.

#### 2.2.5 Visualization Controller

From the scan path followed by the student while moving the probe and from the corresponding practice XML file, the *Visualization Controller* recreates the training replica perceptions on the computer screen. First, the information related to the virtual patient model (*Model* element) and the *Echography* element is extracted and displayed in the two visualization windows, namely, *Virtual Probe* and *Sonoanatomy*, respectively. Next, the *Visualization Controller* visually reproduces the movements performed by the user with the probe replica as on-screen movements of the virtual probe and with the mouse as on-screen movements of the virtual syringe and needle, depending on the simulator, namely, US-DES and US-TS, as explained below.

In the US-DES simulator, the *Visualization Controller* periodically samples the updated pose information of the probe replica at a frequency of 30 Hz, as computed and transmitted by the *Pose Tracking Controller*. Along a *linear scan path*, the real 2D linear coordinates are used to refresh the on-screen position of the virtual probe on the model image in the *Virtual Probe* window. In addition, these linear coordinates serve as an index to select the corresponding US image from the image repository, as defined by the attributes of the *Echography* element for the *linear scan path*. When this index corresponds to the definition of the correct treatment area, the three angular orientation coordinates are used to compute the US image index associated with the *rotation scan path*. Thus, as the user rotates the probe, the angular orientation coordinates are updated, and the corresponding US images are displayed in real time, based on the rotational movements captured by the *Pose Tracking Controller*.

Regarding sonoanatomy, when the US image corresponding to the correct treatment area is displayed, the *Visualization Controller* highlights the interactive contours of the ROIs as the user hovers the mouse over them. These ROIs correspond to the anatomical structures selected by the instructor as relevant for the practice. The attributes for the different *ROIEchography* elements define the polyline coordinates and display format of each ROI, the ROI type (e.g., nerve, vein, muscle), and the anatomical description of the corresponding structure. Similarly, the actual anatomical slice defined in the *AnatomicalSlice* element is displayed in an auxiliary window. The interactive contours associated with the ROIs are also highlighted on this anatomical slice illustration. Using the attributes of the different *ROISlice* elements, the corresponding polylines are visualized according to the defined format, thereby enabling clear correspondence between the anatomical structures shown in the US image and those in the anatomical slice.

For the US-TS simulator, once the correct treatment area is reached by following the scan paths with the replica probe, the *Visualization Controller* displays the US video specified by the *Echography* element. From that moment onward, the movements performed by the student with the computer mouse to modify the syringe orientation and the needle displacement are reproduced in the *Virtual Needle* window.

Regarding the *needle path planning*, the *Visualization Controller* is responsible for geometrically projecting the needle onto the US video displayed in the *Needle Path* window. In addition, the coordinates of the polygonal outlines defined in the *ROIEchography* elements are used. Based on these coordinates, a contact test is performed to determine whether the needle intersects any ROI. Depending on the anatomical structure type, an alert is generated if the needle passes through a forbidden ROI. In this way, the student can plan the *needle insertion path* while accounting for the interactive anatomical contours.

### 2.3 Lumbar facet syndrome treatment simulator

This section presents a complete case study in which the framework elements are applied and specified to develop a training simulator for the treatment of lumbar facet syndrome. It then describes how the resulting simulator operates from an end-user perspective, enabling the practice of sonoanatomy corresponding to a systematic exploration of the lumbar spine and the facet block procedure.

Professional interest in training in ultrasound-guided lumbar spine blocks is hindered by the difficulty of visualizing the spinal sonoanatomy at the lumbar level. Therefore, future specialists need to receive training to acquire skills in identifying the most relevant sonoanatomy of the lumbar spine and in performing systematic procedures for the principal blocks.

The training simulator developed following the proposed framework can support practitioners new to the field in learning to interpret ultrasound images of the lumbar spine and in practicing a systematic approach to performing major lumbar spine blocks. To test the validity of the simulator, the training procedures for lumbar facet joint blocks for the treatment of facet joint (zygapophyseal) syndrome were selected as a case study. As reported in previous studies [29–31], following epidural corticosteroid injection, lumbar facet blocks represent the second most frequently performed interventional procedure for the treatment of chronic pain and were likely the first application in which ultrasound guidance was described for interventional management of lumbar pain.

#### 2.3.1 Visual resources repository for the simulator construction

A team of two healthcare experts participated in the design and development of the case studies. They compiled a set of visual resources such as photographs and US images of a human model. The model was a 46-year-old man with no reported disorders, who was informed about the study objectives and provided written informed consent. The real US images were acquired using an ESAOTE Mylab 25 Gold system equipped with a convex-array transducer (model CA621) operating at of 0.5–1.8 MHz. In addition, US videos demonstrating facet joint infiltration procedures were recorded as audiovisual training resources. This repository was further complemented with illustrations of sectional anatomy slices corresponding to the ultrasound region of interest.

#### 2.3.2 Lumbar spine sonoanatomy practice with the developed simulator

**Figure 3:**
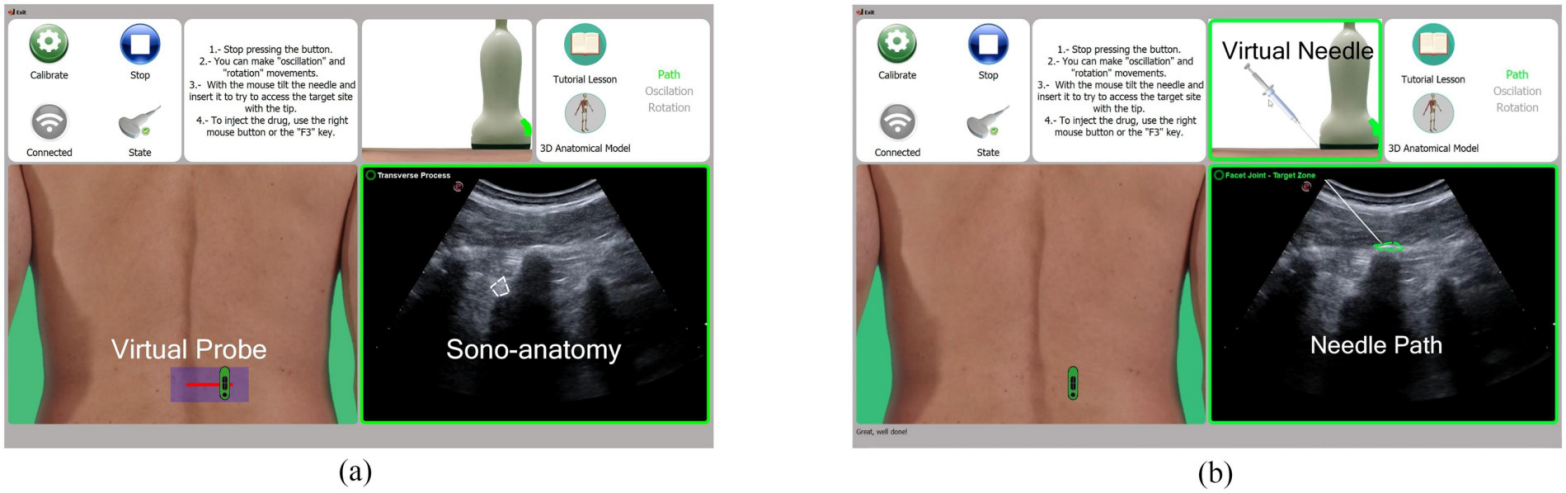
Final views of the US image-based treatment simulator: **(a)** US-DES simulator and **(b)** US-TS simulator.

Before performing any interventional procedure, it is necessary to accurately locate and characterize the lumbar facet joints using ultrasound imaging. Using the US-DES simulator (Figure 3a), students can practice sonoanatomy of the lumbar spine by visualizing US slices acquired initially in sagittal scans and subsequently in transverse scans.

In the US-DES simulator, all resources required for the practice are loaded through the XML file in which the two instructors have defined the practice semantics. In the *Virtual Probe* window, a photograph of the lower lumbar spine of the human model is displayed in the prone position, with the virtual probe initially positioned near the medial region of the spine as the target location. As a learning guide, the predefined *linear scan path* is displayed in red. These resources correspond to the data defined by the instructors within the *Model* element. As the trainee performs linear and angular scan paths with the probe replica, the virtual probe is displayed on the model according to the performed movements. Simultaneously, in the *Sonoanatomy* window, the US slice corresponding to the current linear and angular positions relative to the home reference is shown. These resources correspond to the data contained within the *Echography* element.

The video (https://youtu.be/1tUCXPz8gFU) shows the systematic procedure followed by the students using the constructed training simulator.

#### 2.3.3 Lumbar facet block practice with the developed simulator

After becoming proficient in recognizing the anatomical echo structures of the lumbar spine, the student proceeds to practice the ultrasound-guided infiltration procedure. The US-TS simulator (Figure 3b) incorporates the functionalities of this procedure using a virtual syringe and the replica probe. Also, the visual resources are loaded using the XML file created by the instructors to define the practice semantics.

As the student moves and rotates the probe replica, a color-coded bounding box indicates whether the correct target position is far from, near, or within the correct treatment site (green in Figure 3b). Once this target position is reached, a video is displayed in the *Needle Path* window to provide a realistic moving-image sensation. At this point, the trainee can start practicing with the needle and syringe displayed in the *Virtual Needle* window. Using the mouse, the student can tilt and translate the syringe, while the needle appears in the *Needle Path* window on the US image, with an inclination angle and insertion depth corresponding to the mouse movements.

According to the definitions specified by the instructors in the corresponding practice XML file, the contours of the most relevant echo structures (*ROIEchography*) are highlighted on the US image. In this way the student can identify when either the target structure or a critical structure has been reached with the needle, and interactive text messages are displayed in the *Needle Path* window.

In the video (min 01:09) available at the referenced web link, when the characteristic “double hump” pattern is visualized, the US image is framed with a green rectangle, indicating the correct site for the lumbar facet block. Moreover, the hyperechogenic area corresponding to the facet joint is defined as the target *ROIEchography*, so it is delimited by a green polygonal contour. Conversely, structures such as transverse processes, laminae, vertebral bodies, and vessels are outlined in red to indicate critical structures to be avoided during needle insertion. This visual guidance allows the student to visually check, in real time, whether the planned *needle insertion path* is anatomically feasible and safe.

### 2.4 Training simulator evaluation methodology

This section describes the methodology used to validate the training simulator through an ad hoc questionnaire completed by medical students, focused mainly on the US probe and the graphical user interface. The simulator, incorporating the real haptic probe, was evaluated at the Pain Unit of the Assistential University Complex of Salamanca (Spain). A total of 18 medical students practiced an ultrasound-guided anatomical exploration. All participants voluntarily provided written informed consent prior to their participation, in accordance with ethical requirements for research involving human subjects. The study design was reviewed and approved by the coordination of the Department of Surgery of the Medical Degree at the University of Salamanca. According to institutional guidelines, no formal ethics committee approval was required for this educational study involving anonymous questionnaires and voluntary participation.

The evaluation protocol began with a presentation by an anesthesiologist with more than 30 years of clinical experience, who explained the operation of the application and its GUI, as well as the capabilities of the haptic probe. Subsequently, the objective of the practical exercise was described. Students were instructed to perform a systematic exploration, either in transverse or sagittal orientation, under US guidance until the correct site was found. In both approaches, the students were required to perform the *linear scan path* and *angular scan path* with the probe replica to identify the sono-structures of interest.

Once the medical students had completed the learning experience, they evaluated the simulator using an ad hoc questionnaire designed to assess their perceptions of the experience with the haptic probe. The content of the questionnaire was validated by five experts with more than 15 years of experience in the use of simulators in an educational environment.

The questionnaire was designed to evaluate five specific dimensions: (1) the student profile and prior experience with medical imaging and simulation technologies; (2) perceived learning outcomes and skill acquisition during the training session; (3) the realism and usability of the probe replica, including its external appearance and functionality; (4) the evaluation of the GUI, considering navigability, image quality, and user support; and (5) the overall evaluation of the simulator’s impact on the learning process and user motivation. The responses were collected via online questionnaires, completed anonymously to ensure unbiased feedback. Descriptive quantitative analysis was conducted on the closed-ended responses, while qualitative analysis was applied to the open-ended questions to obtain more detailed insights.

## 3 Results

**Figure 4:**
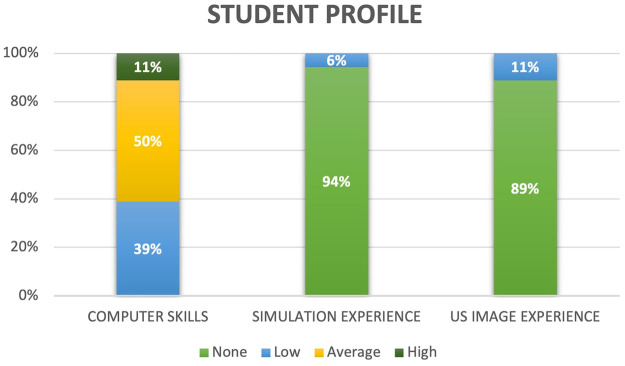
Students previous experience at ICT and simulation.

The results of the students’ evaluations, collected through the questionnaire, are presented according to the five previously defined dimensions. The first dimension (Figure 4) aimed to characterize the profile of the students. The results reflected a moderate level of computer science proficiency, with only one student reporting a single previous medical learning experience in US-based simulation technological environments and a minimal previous experience in US imaging, with only two students having worked one or two times with US imaging.

**Figure 5:**
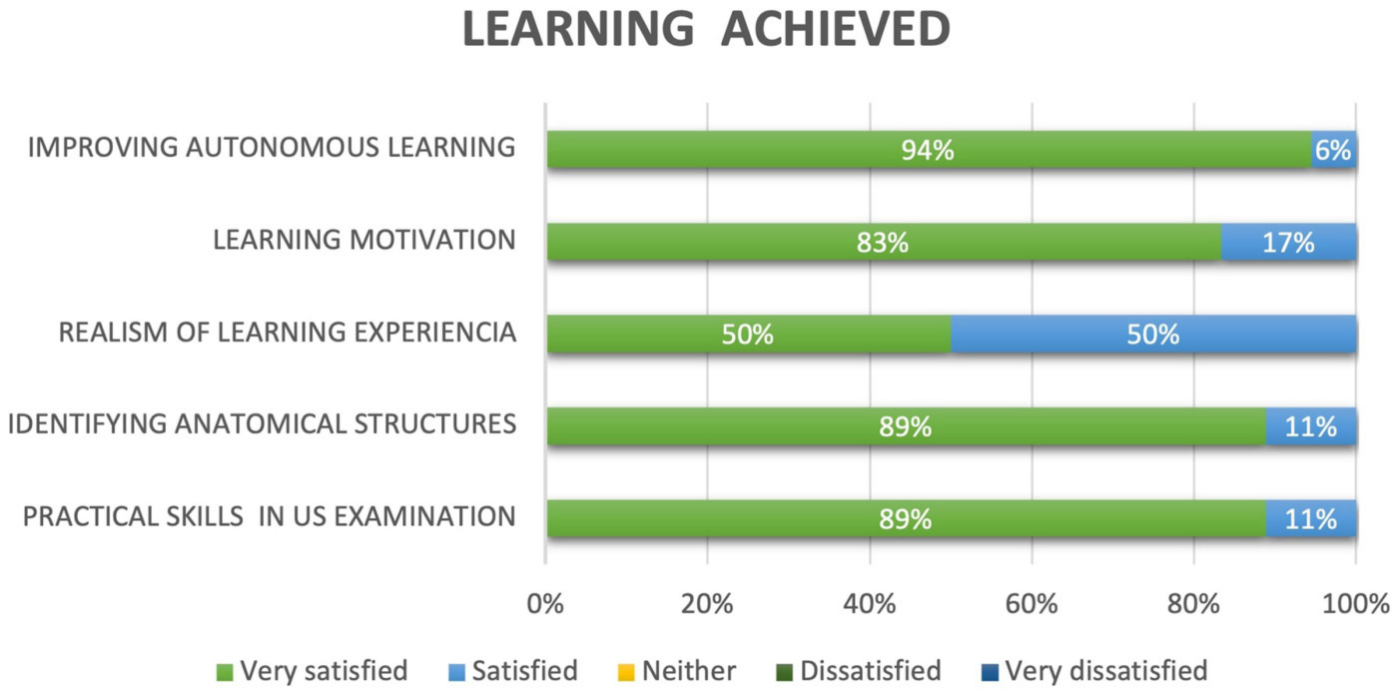
Learning achievement evaluations.

For the second dimension, students were asked to rate the level of learning achieved (Figure 5). In terms of skill acquisition, 89% of the students reported that the simulator accelerated the acquisition of practical skills for the US examination and rated this aspect as *Very satisfied*, while the remaining 11% rated it as *Satisfied*. Similar percentages were obtained for the item related to training and familiarity when identifying the anatomical structures in US images. Both assessments are further supported by the fact that 50% of the students rated the realism of the learning experience as *Very satisfied* and the remaining 50% as *Satisfied*.

Regarding the usefulness of the simulator in increasing motivation to study the presented concepts, 83% expressed a *Very satisfied* opinion, while 17% reported being *Satisfied*. Regarding the overall assessment of the learning process, 94% of the students rated the simulator as *Very satisfied* as an innovative resource that facilitates autonomous training for the studied technique, while the remaining 6% rated it as *Satisfied*.

**Figure 6:**
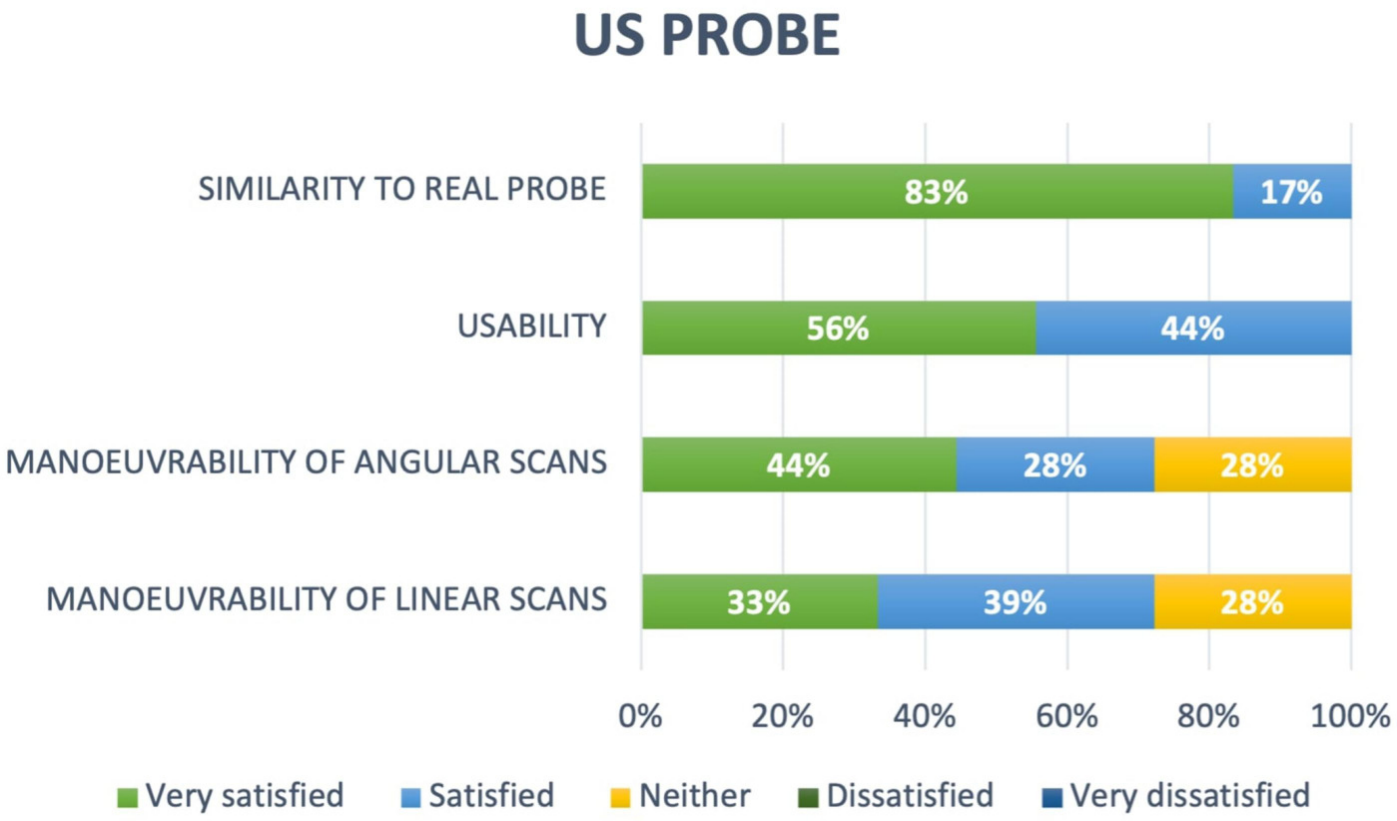
Replica realism ratings.

The third dimension focused on aspects related to the realism of the probe replica (Figure 6). Concerning the similarity between the external appearance of the replica and a real probe, 83% of the students rated it as *Very satisfied*, while the remaining 17% rated it as *Satisfied*. In terms of usability, 56% considered usability of the replica as *Very satisfied*, while the remaining 44% rated it as *Satisfied*. Maneuverability during angular and linear scan path was rated as *Very satisfied* or *Satisfied* by 72% of the students and as *Normal* by the remaining 28%.

**Figure 7:**
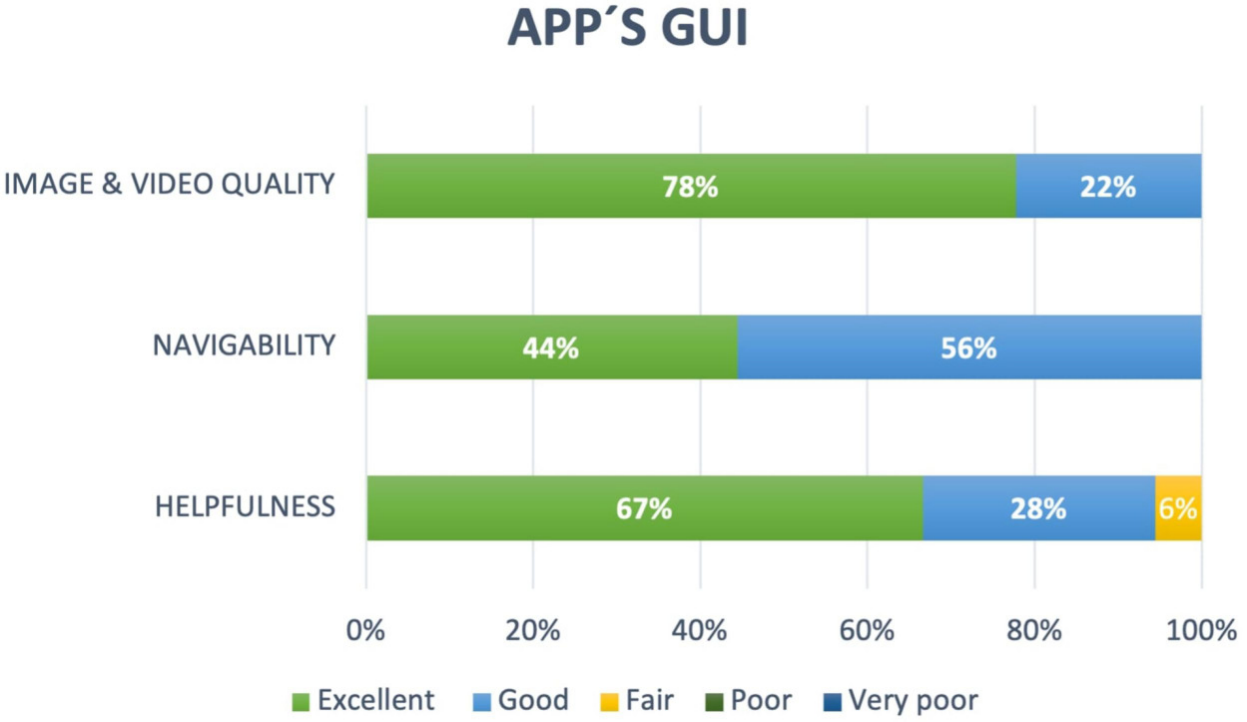
Evaluations of the graphical user interface of the app.

The fourth dimension evaluates the GUI application supporting the learning simulator. As shown in Figure 7, the navigability and image and video quality of the app and the usefulness of the help instructions were rated as *Excellent* or *Good* by most of the students, and only 6% indicated that the help interface was an aspect requiring improvement.

**Figure 8:**
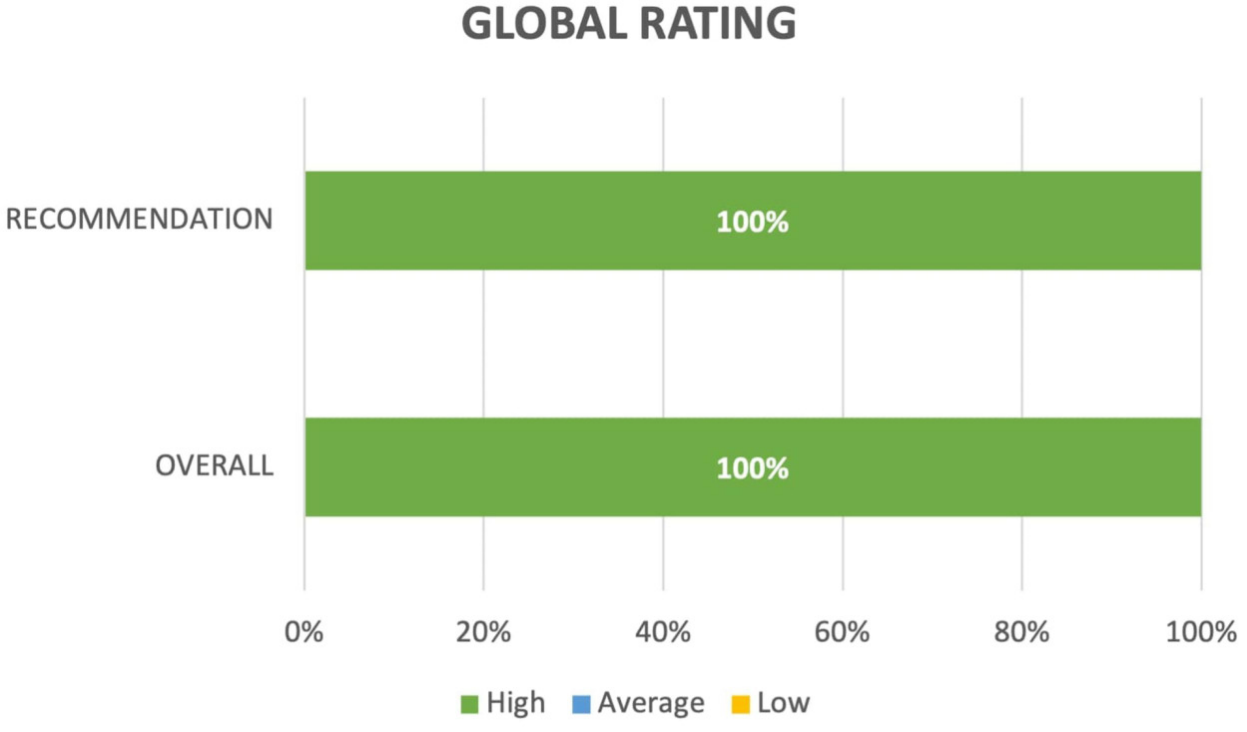
Global simulator performance evaluation.

Regarding the overall rating of this medical training simulator as a whole (Figure 8) and the likelihood of recommending it to a colleague, 100% of the students assigned the highest rating.

To allow students to express their opinions freely, two open-ended questions were included. Regarding the first open-ended question, “What aspects of this learning experience do you consider positive or remarkable?,” the most representative student responses highlighted several key advantages of the simulator:

Possibility of repeated practice without requiring a model or patient: “Working with a machine allows you to repeat scans as many times as you want without requiring the collaboration of a model or patient.”
Reduction of performance pressure: “To be able to practice alone without the pressure of doing it in front of a doctor hurrying or in front of a patient.”
Realism of the equipment: “To be able to learn with equipment similar to the real thing.”
Realism of the visualization: “The fact that it represents what you will see is very helpful in preparing you for the real situation.”
Fidelity of the simulated exploration: “Simulation approximates the structures and exploration reality.”
Improved familiarity with ultrasound interpretation and probe handling: “I consider it useful to be able to handle a simulator prior to being with a patient to become familiar with the visualization of the ultrasound image and the operation of the probe simultaneously.”
Effective first contact with US imaging: “Very useful as a first contact with echography.”
Reinforcement of theoretical knowledge: “To be able to review the knowledge we have been taught in the classroom by means of the simulator, being able to do it in depth.”
Facilitator of practical learning: “More practical learning.”

Regarding the second open-ended question, “Which aspects from this learning experience do you see as negative and possible improvements?,” the following responses are highlighted:

Restricted visualization range: “Only images of the areas within the path are visible.”
Need for additional US images from adjacent regions: “Addition of images from adjacent areas, to make it more difficult to locate the study area”; “I would add more US slice on each of the explorations as well as out-of-area images to get a more close-to-reality experience.”
Limited tactile feedback due to the absence of a real patient: “The fact that it’s not a real person in some ways it limits what you can visualize and the overall experience. You lose the pressure and the reliefs.”
Improvements in probe interaction: “The sensitivity of the probe needs to be improved, but in general the response is good”; “I would like the probe to detect pressure, I know it’s a complex one.”
Improvements in usability: “The scroll pad could be a little larger for more comfort.”
Expansion of training content: “That more anatomical areas can be practiced in the future”; “I hope that their use can be extended to improve the learning process of ultrasound techniques.”

Despite these limitations and suggestions for improvement, students expressed strong support for the continued development and broader adoption of the simulator “I sincerely believe that this type of learning is much more useful than the theoretical, so I encourage you to continue progressing and improving the technology and applying it to all possible areas” and emphasized its potential curricular value “They should supplement the radiology course with this type of simulator. That would be a great end.”

## 4 Discussion

This section provides a discussion of the presented work from two complementary perspectives: that of the student, emphasizing usability, perceived learning outcomes, and learning experience realism, and that of the instructor, focusing on training design, case preparation, and skill acquisition.

### 4.1 From the student experience point of view

The evaluation results indicate that the proposed simulator is a useful training tool for US-guided procedures, with students reporting a positive reception in terms of usability and learning support. High levels of student satisfaction across all five evaluated dimensions demonstrate its capacity to facilitate cognitive learning, skill acquisition, and student motivation and participation. In particular, the realism of the probe replica, the quality of the GUI app, and the guided learning process were identified as key factors contributing to a positive learning experience. The qualitative feedback further supports these findings, highlighting the benefits of repeated autonomous practice, reduced procedural stress for students when practicing under instructor supervision or with a patient, and improved familiarity with US image interpretation and probe handling. Although certain limitations were identified, such as restricted tactile feedback and limited visualization range, these aspects were perceived as opportunities for future enhancement rather than barriers to learning. Overall, the simulator was strongly valued as a complementary training resource with high potential for integration into medical curricula, reinforcing its role as a valuable bridge between theoretical instruction and real clinical practice.

It should be noted that the evaluation focuses on learner perceptions and training support rather than on direct clinical performance or comparative assessment with other training modalities. Consequently, the reported findings should be interpreted within a formative and educational context, rather than as evidence of clinical equivalence with real US-guided procedures.

Previous studies have explored a wide range of training modalities for US-guided procedures, each emphasizing different aspects of the learning process. Tissue-simulating phantoms [18] primarily focus on reproducing the acoustic properties of biological tissues, offering high realism in image formation but limited flexibility and durability. Purely software-based simulators and web-based platforms [22] emphasize accessibility and scenario variability, often at the expense of realistic probe handling and reproduction of clinical gestures. More immersive approaches based on virtual environments [32] prioritize visual immersion and spatial understanding, while mechanical haptic interfaces provide precise motion tracking and force feedback but may constrain natural probe manipulation. In contrast, the approach presented in this work is positioned as a solution that prioritizes preservation of the clinical gesture and brain–hand–eye coordination, while maintaining flexibility in case definition and scenario design.

### 4.2 From the teaching experience point of view

There is no doubt that the success of a simulator largely depends on the quality of the learning resources and the scope of the training cases [33].

With regard to the learning resources, a fundamental requirement is the involvement of clinical specialists who provide expert knowledge to construct high-quality practical training cases, as well as the theoretical tutorial content supported by multimedia resources. When incorporating new training case studies, instructors must address the cumbersome task of preparing appropriate learning resources. Among these, a database of US images related to the case studies should be available and a clear correspondence between structures in ultrasound images and those in real anatomical illustrations must be established.

In this project, we employed one of the most common and realistic method for simulating 2D US images by interpolating 3D US volumes previously acquired from real subjects. Once the US videos are recorded, it is necessary to filter the frames, as real-time exploration may yield some frames that are not suitable for constructing the intended practice. In addition, these resources consume a significant disk space, requiring instructors to balance image quality and storage efficiency. This process involves selecting frames that accurately represent key anatomical structures while ensuring that the dataset remains manageable in size for practical use in educational environments.

Recording the US image database and establishing the geometric correspondence [33] between the US images and the points along the linear and angular scan paths in the patient coordinate system remain time-consuming and cumbersome tasks that must be performed by the instructor. By using XML together with the developed application *Case Training Manager*, the construction of case studies within the framework is significantly facilitated and made more flexible. A potential solution to further simplify the work of the instructor in constructing the training cases might be to use synthetic US images generated by computational models that simulate ultrasound wave propagation phenomena (reflection, refraction, scattering, and absorption) within a 3D anatomical graphical representation. Moreover, variations in parameters associated with the ultrasound scanner, such as frequency, depth, gain, and focus, can be incorporated in these synthetic US images, which is not feasible in a real US repository due to the significant recording effort involved. Nevertheless, the proposed framework is equally applicable to repositories of synthetic US images generated by these methods when applied to educational medical databases available, such as Visible Human, or synthesized from clinical databases from other medical imaging sources such as CT or MRI.

The proposed simulator has been designed by medical experts, which has been crucial for ensuring a realistic training experience across key aspects of the design, particularly in the design of the human–machine interface, which is fundamental for effective visual feedback, and in the sensitivity configuration of the probe replica to achieve true-to-life behavior.

It is also essential that the experts evaluate the simulator in terms of the fidelity to the learning experience. In this study, qualitative feedback was collected from seven experts through oral interviews. In all cases, the experts expressed high satisfaction with the realism of the learning experience and its strong applicability as a complementary training resource for acquiring the skills needed for US image-based diagnostics, clearly emphasizing the need to practice on a real ultrasound scanner. Nevertheless, future work should focus on obtaining more objective and quantitative expert assessments, as stated in [34]. The experts also highlighted the importance of incorporating a real needle into the simulator to achieve a more realistic sensation in interventional procedure training. This represents an ongoing research direction that we plan to address in the near future, thanks to the expertise gained in the probe attitude estimation using the developed US replica.

In line with this need for rigorous assessment, it is important to note that the evaluation conducted with students focused solely on the US-guided simulator, not the underlying framework. While a qualitative evaluation by experts was performed, as previously described, a formal evaluation methodology, such as Design Science Research, was not used. The expert validation provided useful insights, but the lack of a structured, formal evaluation approach limits the ability to assess the overall effectiveness of the framework. Future work should apply more rigorous methodologies to evaluate both the simulator and the framework.

## 5 Conclusions

This work presents the design, implementation, and validation of a training simulator for ultrasound-guided lumbar facet syndrome treatment, developed within a modular learning framework aimed at supporting the construction of procedure-specific simulators. The proposed solution integrates a realistic probe replica with a desktop-based visualization system, enabling trainees to practice probe manipulation, sonoanatomy interpretation, and interventional planning in a controlled and repeatable environment.

The evaluation conducted with final-year medical students indicates a high level of acceptance in terms of usability, realism, and perceived learning support, suggesting that the simulator enables repeated autonomous practice, reduces procedural stress when students subsequently perform techniques under instructor supervision or with real patients, and improves familiarity with ultrasound image interpretation and probe handling. Student feedback further confirms that the simulator constitutes a valuable educational tool for introductory and complementary training in ultrasound-guided procedures. These results support the suitability of the proposed approach for facilitating the acquisition of essential cognitive and motor skills prior to clinical practice.

Despite these promising results, several aspects remain open for further development, including the systematic evaluation of the underlying framework using formal methodologies, the incorporation of objective performance metrics, the integration of additional sensory feedback into the probe replica, and the incorporation of a sensorized real needle. Addressing these aspects will strengthen both the educational impact and the general applicability of the proposed framework.

## Data Availability

The original contributions presented in the study are included in the article, further inquiries can be directed to the corresponding author.
